# Depression and Associated Factors among Family Caregivers of Children with Disabilities: Analysis of Intergenerational Differences

**DOI:** 10.3390/healthcare11192693

**Published:** 2023-10-08

**Authors:** Cong Xia, Ting Wei, Qi Tang, Hongying Zheng, Gang Chen, Jun Lu

**Affiliations:** 1 School of Health Management, Anhui Medical University, Hefei 230032, China; xiacong@ahmu.edu.cn; 2 School of Public Health, Fudan University, Shanghai 200032, China; 21111020078@m.fudan.edu.cn (T.W.); tangqi@fudan.edu.cn (Q.T.); 20111020062@fudan.edu.cn (H.Z.); 3 China Research Center on Disability, Fudan University, Shanghai 200032, China

**Keywords:** children with disabilities, depression, family caregivers, modifiable factors

## Abstract

Family caregivers of children with disabilities might face high risks of depression, whereas the existing literature focused more on parents neglecting grandparents. This study investigated 380 parents and 108 grandparents of children with disabilities to identify depression and associated factors. Descriptive statistics, Chi-square test, Mann–Whitney U test, and multivariable logistic regression were performed to describe the participants’ characteristics and risks of depression and identify significant factors. Results showed that parents (35.5%) had higher risks of depression than grandparents (32.4%), but statistical differences were not found. Children’s sleep problems (AOR = 1.751, 95%CI = 1.019, 3.008), harmonious family relationships (AOR = 0.694, 95%CI = 0.569, 0.846), and better barrier-free construction (AOR = 0.742, 95%CI = 0.568, 0.970) were significantly associated with depression among parents. As for grandparents, higher education (AOR = 4.108, 95%CI = 1.526, 11.057) and caring for children who experience frequent mood swings (AOR = 2.242, 95%CI = 1.161, 4.329) were associated with higher risks of depression. Further, house ownership (AOR = 0.167, 95%CI = 0.031, 0.887), higher family cohesion (AOR = 0.545, 95%CI = 0.297, 1.000), and better barrier-free construction (AOR = 0.401, 95%CI = 0.185, 0.869) were associated with lower odds of depression. Therefore, both parents and grandparents of children with disabilities had high risks of depression and thus required urgent attention. Healthcare providers and policymakers should develop and implement interventions considering intergenerational differences to reach optimal efficiency.

## 1. Introduction

Depression is one of the most frequently occurring psychological disorders, with an estimated 5% of adults suffering from depression [1]. It is a leading cause of disability worldwide and a major contributor to the global disease burden. Depression is different from usual mood fluctuations and is characterized by the presence of sadness, irritability, emptiness, loss of interest, excessive guilt, low self-worth, hopelessness about the future, or even suicide [1,2].

According to the World Health Organization (WHO), children with disabilities is defined as the umbrella term referring to children who have impairments, activity limitations, and participation restrictions [3]. The number of children with disabilities globally is estimated at almost 240 million, according to a new UNICEF report [4]. Additionally, the number in China in 2006 was about 5 million [5]. Nowadays, although more and more children are surviving, the number of children with disabilities is increasing [6]. Children with disabilities require more health and other related services than typically developing children, such as rehabilitation and long-term care, which puts higher requirements on family caregivers [7]. A family caregiver of children with disabilities typically refers to the parent (either mother or father) or other family members (grandmother or grandfather, siblings, et al.) who are responsible for the assistance and comprehensive care of children. Researchers have found that depression is associated with high stress [8]. Raising children with disabilities means a series of stressors for family caregivers, such as children’s movement disorders [9], emotional and behavioral problems [10], stigma [11], long-term and high-intensity caregiving [12], and financial strain [13]. Hence, this population may be more vulnerable to depression and need to be concerned [14].

Fortunately, many researchers have paid attention to the risk of depression among family caregivers, especially parents of children with disabilities (e.g., Cerebral Palsy (CP), Autism Spectrum Disorder (ASD), Down syndrome), and concluded a higher prevalence of depression than among parents of typically developing children [15,16]. A systematic review analyzed 19 studies across 11 countries, including 3303 parents of children with intellectual and developmental disabilities (IDD) and 9519 parents of children without IDD [17]. Results showed that, on average, 31% of parents of children with IDD suffered from moderate depression compared to 7% of parents of children without IDD. Based on the evidence, researchers have also explored factors associated with parents’ depression of children with disabilities and proposed various targeted interventions, such as the Group Stepping Stones Triple P training program, Mindfulness-Based Stress Reduction, Training and Support Program, and Acceptance and Commitment Therapy.

Nowadays, besides parents, grandparents are also actively involved in caring for grandchildren, especially those with disabilities. As mentioned above, a child’s disability places tremendous pressure on parents, and in order to mitigate the negative effects, grandparents often take on the responsibility of caring for their grandchild [18]. Grandparents provide emotional, financial, and instrumental support for their adult children and grandchildren. Specifically, they encourage their adult children to be optimistic [19], give out their surplus funds to help cover family costs [20,21], and assist with babysitting [22], transportation [23], and housework tasks [24]. Such experience could also place grandparents at risk of depression, but the associated research is limited. A scoping review of the literature on grandparents of children with disabilities identified 31 studies and grouped findings into adjustment to the grandchild’s disability, roles, perceptions, and experiences, without any report of the depression status [25]. This situation resulted in sparse knowledge on the impact of caring for children with disabilities on grandparents’ depression and the corresponding improvement measures. The interventions obtained from existing literature focused on parents cannot be directly applied to grandparents because of the heterogeneity of the two populations.

According to the International Classification of Functioning, Disability and Health (ICF) and the International Classification of Functioning, Disability and Health for Children and Youth (ICF-CY), the health of both children and family caregivers is influenced by the children’s and family caregivers’ characteristics as well as the contextual factors shared by them [3]. That means children’s disability might impact family caregivers’ depression, and caregivers’ depression could also affect children’s health development. Consequently, there is a need to investigate depression and associated factors of grandparents alongside parents. Additionally, the findings on intergenerational differences can help guide more precise interventions. If parents and grandparents are exposed to depression for a long time without any change, the negative impact would further decrease the family’s quality of life and increase the social burden.

ICF contains two parts; the second part (contextual factors) involves environmental factors and personal factors, providing a framework for comprehensively identifying factors that are associated with family caregivers’ depression [3]. In this study, the potential factors were categorized into four aspects: child, caregiver, family, and social-related factors [3]. International literature has also provided a substantial amount of empirical evidence. However, the relationship applied to grandparents and parents of children with disabilities is unclear. From a service perspective, it is crucial to identify modifiable factors so that evidence can support developing effective strategies to promote their mental health [26]. Given this, this study aimed to investigate the current situation of risks of depression among grandparents compared to parents and identify the associated modifiable factors. Our research questions were:

1. Is the risk of depression of grandparents different from that of parents?
2. Is the relationship between the risk of depression and modifiable factors among grandparents different from parents?

## 2. Materials and Methods

### 2.1. Participants

We recruited family caregivers of children with disabilities in Shanghai from July to August 2020 and in Kunshan from November to December 2021. The cross-sectional investigation was conducted in 8 rehabilitation institutions in Shanghai and two rehabilitation institutions in Kunshan using convenience sampling method. In each institution, parents or grandparents were included if (1) they were caring for children diagnosed with one or more kinds of disability aged <18 years (e.g., speech disability, intellectual disability); (2) they were primary family caregivers who took care of children for more than 40 h per week; (3) they had no communication barriers and consented to participate in this investigation.

The investigation procedure was as follows: First, the researchers explained the purpose and significance of the study to the participants. Then, they briefly introduced the questionnaire content to participants and how to fill it out. For illiterate participants, researchers read the questions and made choices based on the participants’ answers. Questionnaires were collected by the researchers directly after completion. Finally, 488 family caregivers were recruited, with 327 from Shanghai and 161 from Kunshan. Specifically, 306 mothers, 74 fathers, 70 grandmothers, and 38 grandfathers participated in the survey, respectively. As for the disability types of their children, 101 children were diagnosed with hearing and speech disability, 14 children were diagnosed with vision disability, 96 children were diagnosed with physical disability,47 children were diagnosed with intellectual disability, 79 children were diagnosed with mental disability, and 151 children were diagnosed with multiple disabilities. Ethical approval was obtained from the Ethics Committees of the School of Public Health Fudan University (IRB No.: 2019-10-0782).

### 2.2. Measures

#### 2.2.1. Depression

Depression was measured using the Patient Health Questionnaire-9 (PHQ-9), which was developed by Kroenke in 2001, considering its time efficiency and precision [27]. PHQ-9 consists of nine items and is a brief self-report scale to make criteria-based diagnoses of depression. Each item asks the frequency they have been bothered by the specific symptom over the last 2 weeks, scoring 0–3. Total PHQ-9 scores range from 0 to 27, with higher scores indicating greater depression. Cut points 5, 10, 15, and 20 represent mild, moderate, moderately severe, and severe depression, respectively. Compared with other longer measures, PHQ-9 has good sensitivity and specificity and is more time-efficient; it has been translated into more than 100 languages and is widely used to screen for depression among various populations (e.g., adults with epilepsy, patients with coronary artery disease, and mothers of children with neuro-developmental disorders) [28,29,30]. In 2014, the Chinese version of the PHQ-9 was validated in 1045 residents from a Shanghai community, and results showed that PHQ-9 is an appropriate and efficient tool for screening depression in the general Chinese population [31]. In this study, we used the Chinese version of the PHQ-9 to screen depression in family caregivers of children with disabilities, and a score of 5 is the cutoff point. Among the 488 participants, Cronbach’s α was 0.889, χ2/df = 1.463, Goodness of Fit Index (GFI) = 0.989, Comparative Fit Index (CFI) = 0.997, Normed Fit Index (NFI) = 0.989, Incremental Fit Index (IFI) = 0.997, Root Mean Square Error of Approximation (RMSEA) = 0.031, indicating good reliability and validity.

#### 2.2.2. Associated Factors

We identified the associated factors from four aspects: child, caregiver, family, and social-related factors. Specifically, child-related factors examined demographic characteristics of physical, mental, and social health, including age, gender, disability type, disability severity, comorbidities, sleep problems, mood swings, home participation, and community participation. According to the People with Disabilities Act of the People’s Republic of China, disability is divided into visual disability, hearing disability, speech disability, physical disability, intellectual disability, mental disability, multiple disability, and other disabilities. Caregiver-related factors included age, gender, marital status, education, employment status, and comorbidities, reflecting both demographic characteristics and health status. Family-related factors were measured from the hard and soft environments, including family income, house ownership, and family cohesion. As for social-related factors, we examined rehabilitation assistance, policy satisfaction, social attitude, and barrier-free construction. Among these associated factors, the caregiver’s age, gender, child’s age, gender, and disability type were non-modifiable factors and, hence, were identified as control variables.

### 2.3. Statistical Analyses

The coefficient of Cronbach’s α and confirmatory factor analysis were used to assess the reliability and validity of PHQ-9 in family caregivers of children with disabilities. The Chi-square test and Mann–Whitney U test were used to identify the differences in sociodemographic characteristics between parents and grandparents. To examine the associations between the risks of depression and child, caregiver, family, and social factors, we conducted multivariate logistic regression by controlling non-modifiable factors. Adjusted odds ratios (AORs) and 95% confidence intervals (CIs) were presented in Model 1 and Model 2. A two-tailed *p* < 0.05 was considered statistically significant. Statistical analyses were performed using SPSS 25.0 and AMOS 24.0.

## 3. Results

### 3.1. Sociodemographic Characteristics

Among 488 family caregivers who responded to the survey, parents comprised 77.9% and grandparents 22.1%. For the whole sample, 64.1% of the children they cared for were boys, and 30.9% were diagnosed with multiple disabilities. The sociodemographic details are displayed in Table 1. Compared with parents, grandparents were significantly less educated, more unemployed, had a higher proportion of males, and a higher risk of comorbidities (*p* < 0.05). As is shown in Table 1, 35.5% of parents had the risk of depression, and the rate among grandparents was 32.4%, without a statistically significant difference.

### 3.2. Risks of Depression

Table 2 reports the risk of depression among family caregivers of different caregiving roles. Of the total sample, 34.8% were screened for depression, and 11.1% were screened for moderate to severe depression. Among parents, 11.3% were screened for moderate to severe depression, and the rate among grandparents was 10.3%. In general, mothers (38.2%) had a higher risk of depression than fathers (24.3%), and the risk of depression of grandmothers (38.6%) was higher than grandfathers (21.1%).

### 3.3. Modifiable Factors Associated with Risks of Depression

In Model 1, caregiver-related factors were found to have no significant impact on the risk of depression (see Table 3). Parents caring for children with sleep problems were more likely to get depressed (AOR = 1.751, 95%CI = 1.019, 3.008). In terms of environmental factors, parents who lived with harmonious family relationships had significantly lower risks of depression (AOR = 0.694, 95%CI = 0.569, 0.846). The better the barrier-free construction is, the less likely for parents to get depressed (AOR = 0.742, 95%CI = 0.568, 0.970).

In Model 2 (see Table 3), grandparents with higher education (AOR = 4.108, 95%CI = 1.526, 11.057) and caring for children who experience frequent mood swings (AOR = 2.242, 95%CI = 1.161, 4.329) were associated with a higher risk of depression. Further, the likelihood of depression among grandparents was also found to be negatively impacted by house ownership (AOR = 0.167, 95%CI = 0.031, 0.887), higher family cohesion (AOR = 0.545, 95%CI = 0.297, 1.000), and better barrier-free construction (AOR = 0.401, 95%CI = 0.185, 0.869).

For both parents and grandparents, family cohesion and barrier-free construction were significantly associated with the risk of depression (*p* < 0.05). Nevertheless, the intergenerational difference was found in the relationship between the risk of depression and caregivers’ education, children’s sleep problems, mood swings, and house ownership.

## 4. Discussion

To the best of our knowledge, this is one of the very few studies that examined the modifiable factors associated with depression from four aspects: child, caregiver, family, and society. Further, this is also the first study to investigate depression and associated factors from the perspective of intergenerational differences.

In this study, we found that 35.5% of parents had risks of depression, higher than that among the general Chinese population (26.9%) [31]. This prevalence is also consistent with a systematic review of depression in parents of children with IDD [17], indicating the necessity to pay more attention and provide effective interventions. Among grandparents, 32.4% of them were screened for depression, with no significant difference from parents. Our data show that not only the depression of parents but also grandparents should be urgently concerned. In keeping with previous literature, mothers experienced higher risks of depression than fathers [14], and the same finding was obtained among grandparents. Possible explanations for this may be that (1) female caregivers take on more caregiving responsibilities and practical household chores (e.g., cooking, washing, cleaning) [32]; (2) female caregivers are more likely to give up their jobs, interests, and social networks to care for children’s daily lives and rehabilitation service utilization [14,33]; (3) male caregivers tend to underreport depressive symptoms when using self-report measures [34]. In order to better promote the mental health of parents and grandparents, attention should therefore shift towards the service provision considering gender differences.

Parents caring for children with sleep problems were more likely to get depressed, whereas grandparents caring for children who experience frequent mood swings were associated with higher risks of depression. Sleep problems are common in children with disabilities. In our study, 30.9% of children with disabilities reported sleep problems, such as somnolence, difficulty falling asleep, night waking, and dreaminess. Richdale et al. [35] reported that 58.6% of children with an intellectual disability have a sleep problem. Sleep problems are also likely to be stressful for other family members, particularly parents. Typically, children sleep with their parents or are checked by their parents at night. For children with disabilities experiencing sleep problems, their parents check more frequently, and the sleep quality is even less optimistic. Previous studies have proven that the majority of people with depression have sleep problems, and those with sleep problems have a significantly higher risk of developing depression than those without sleep problems [36]. Therefore, parents with children experiencing sleep problems might be more likely to experience depression but still need further confirmation. Wayte et al. [37] identified that children with CP had higher rates of sleep problems than typically developing peers. Additionally, about 40% of mothers of children with CP reported sleep disorders, of whom 44% had depressive symptoms, suggesting that the sleep disruption in children may be causally related to maternal sleep problems, which in turn may contribute to maternal depression [37]. Children with disabilities, especially children with ASD, are often recognized for their emotional problems (e.g., tantrums and meltdowns), which are inextricably linked with undesirable behavioral problems [38]. Therefore, emotion management strategies or emotion regulation interventions may be given to children if they throw tantrums frequently, which can further alleviate the depression level of grandparents.

No significant caregiver-related factor was found among parents, and only education was identified as associated with grandparents’ depression status. As primary caregivers, they sacrifice almost 24 h a day carrying out responsibilities of children’s long-term rehabilitation, coordination of health service delivery, and assisting with daily activities [39]. During the interviews conducted by our research group, both parents and grandparents reported the highest need for children’s rehabilitation services utilization but the least need for their health management. This finding reflects the low priority parents and grandparents place on their health status, so practitioners should be active in parent education or parent training services.

Better family cohesion was associated with lower risks of depression among both parents and grandparents, which is in line with the relationship found in previous literature [40]. Although grandparents have some different experiences of adjusting to a child’s disability than parents do, several studies showed similar adjustment: initial shock and denial→adjustment and acceptance→settlement and coping [41]. According to family ecosystem theory, each member in the family system has a role and is critical to support other family members when dealing with stressful situations [42]. In a family with better cohesion, family members would understand, encourage, and support each other [43]. Nevertheless, a family with worse cohesion may be full of blame and complaints, increasing the risk of depression [11].

However, different from the findings of previous studies [44,45], we found no significant impact of family income on the risks of depression among both parents and grandparents. This interesting finding may be related to the unique welfare policy in China. In 2018, the State Council issued Opinions on Establishing a Rehabilitation Assistance System for Children with Disabilities, ensuring basic rehabilitation services for children with disabilities, and alleviating the financial burden of families of children with disabilities [46]. Shanghai and Kunshan are two cities with a high amount of rehabilitation assistance. Take children with ASD as an example. In Shanghai, every child with ASD aged 0–18 could receive 24,000 yuan per year. In Kunshan, each child with ASD aged 0–6, 7–14, and 15–18 years old could receive 40,800, 34,800, and 18,000 yuan per year, respectively. In many countries, young people prefer renting to buying their homes [47], but older people still believe owning a house makes you feel like you belong, which may be why living in rented houses is associated with higher risks of depression among grandparents.

Barrier-free environment construction is an important sign of a country’s level of civilization and is necessary for the social participation of children with disabilities [48]. In this study, we found better barrier-free construction was associated with lower risks of depression among both parents and grandparents, demonstrating family caregivers’ focus on an accessible environment for their children or grandchildren. In China, the barrier-free environment construction system has made great achievements since the legislation of barrier-free environment construction [49]. However, there is still a big gap between the current situation and the requirements of family caregivers. Many problems remain, including low awareness, insufficient supervision, and inappropriate design [50]. Therefore, relevant government departments should establish management measures to solve these problems and protect the rights of children with disabilities practically.

There are some limitations in our study. First, our findings described the current situation grandparents are faring compared to parents. There is a selection problem. Some families are selecting their grandparents to be caregivers, and some are not. The things that influence whether grandparents are selected as caregivers may also influence their likelihood of being depressed. The results, therefore, may be biased estimates of what would happen to grandparents’ well-being if there was some exogenous shock (e.g., a policy change that increased or decreased the need for grandparent caregivers). Consequently, further research could be done as to what are the things that lead grandparents to be caregivers. Second, different survey methods for illiterate and literate participants can have an impact on the results. Additionally, our study did not include a comparison group that was administered the survey at the same time in the same way, which would lead to measurement error. Hence, we should refine the study design and survey method to minimize possible measurement errors. Third, we would not infer causal associations due to the cross-sectional design. Further research may be conducted to identify causal relationships between significant factors found in this study and depression. Further, given the small sample size, we did not analyze the factors influencing the risk of depression of mothers, fathers, grandmothers, and grandfathers separately, and it causes difficulties in identifying the determinants of parents’ or grandparents’ depression of children with a specific disability. Therefore, further research should expand the sample size and segment the study population.

## 5. Conclusions

In summary, this study used PHQ-9 to assess the risk of depression and investigated intergenerational differences in associated modifiable factors among parents and grandparents of children with disabilities. Our main findings were: (1) both parents and grandparents had high risks of depression, and (2) intergenerational difference was found in significant modifiable factors affecting the risk of depression in parents and grandparents. Therefore, healthcare providers and policymakers need to emphasize the importance of mental health and take measures to alleviate the depression of this population. Additionally, our findings could provide references for further research to identify causal relationships between these significant factors and depression among this population.

## Figures and Tables

**Table 1:** Sociodemographic characteristics (*n* = 488).

	Parents		Grandparents		Statistics	*p*
	*n*	%	*n*	%		
Gender						
Male	74	19.5	38	35.2	11.740 ^a^	<0.001
Female	306	80.5	70	64.8		
Age (Mdn, IQR)	(36.35, 6.81)		(60.85, 10.64)		−15.586 ^b^	<0.001
Marital status						
Married	368	96.8	102	94.4	0.770 ^a^	0.380
Single/divorced/windowed	12	3.2	6	5.6		
Education						
Junior high school and below	25	6.6	57	52.8	167.855 ^a^	<0.001
Senior high school	68	17.9	36	33.3		
Junior college and above	287	75.5	15	13.9		
Employment status						
Not employed	156	41.1	98	90.7	83.192 ^a^	<0.001
Part-time/full-time employed	224	58.9	10	9.3		
Comorbidities						
No	338	88.9	41	38.0	126.019 ^a^	<0.001
Yes	42	11.1	67	62.0		
Gender of children						
Male	250	65.8	63	58.3	2.033 ^a^	0.154
Female	130	34.2	45	41.7		
Age of children (Mdn, IQR)	(5.13, 3.23)		(5.32, 2.69)		−0.078	0.938
Disability type of children						
Hearing&speech	82	21.6	19	17.6	9.564 ^a^	0.089
Vision	10	2.6	4	3.7		
Physical	68	17.9	28	25.9		
Intellectual	36	9.5	11	10.2		
Mental	70	18.4	9	8.3		
Multiple	114	30.0	37	34.3		
Depression						
PHQ-9 score <5	245	64.5	73	67.6	0.360 ^a^	0.548
PHQ-9 score ≥ 5	135	35.5	35	32.4		

Mdn: Median. IQR: Interquartile Range. ^a^ Chi-square Test. ^b^ Mann–Whitney U Test.

**Table 2:** Risks of depression (*n*/%).

	Minimal	Mild	Moderate	Moderately Severe	Severe
Mothers (*n* = 306)	189 (61.8)	76 (24.8)	26 (8.5)	12 (3.9)	3 (1.0)
Fathers (*n* = 74)	56 (75.7)	16 (21.6)	0 (0.0)	1 (1.4)	1 (1.4)
Grandmothers (*n* = 70)	43 (61.4)	17 (24.3)	7 (10.0)	2 (2.9)	1 (1.4)
Grandfathers (*n* = 38)	30 (78.9)	7 (18.4)	0 (0.0)	0 (0.0)	1 (2.6)
Total	318 (65.2)	116 (23.8)	33 (6.8)	15 (3.1)	6 (1.2)

The total percentages of some variables are not equal to 100 due to rounding.

**Table 3:** Associations between the risk of depression and modifiable factors.

	Model 1: Parents		Model 2: Grandparents	
	AOR	95%CI	AOR	95%CI
Child-related				
Disability severity	0.983	(0.771, 1.253)	0.680	(0.357, 1.293)
Child’s comorbidities	1.428	(0.738, 2.763)	0.357	(0.074, 1.727)
Sleep problem	1.751 *	(1.019, 3.008)	1.505	(0.368, 6.159)
Mood swing	1.160	(0.907, 1.484)	2.242 *	(1.161, 4.329)
Home participation	0.929	(0.777, 1.110)	0.874	(0.538, 1.418)
Community participation	0.807	(0.644, 1.011)	1.351	(0.761, 2.400)
Caregiver-related				
Marital status	0.648	(0.139, 3.015)	0.071	(0.002, 2.331)
Education	1.130	(0.730, 1.750)	4.108 **	(1.526, 11.057)
Employment status	0.622	(0.359, 1.077)	0.346	(0.019, 6.250)
Caregiver’s comorbidities	1.302	(0.589, 2.876)	2.165	(0.573, 8.173)
Family-related				
Family income	0.874	(0.685, 1.114)	0.525	(0.252, 1.091)
House ownership	0.573	(0.307, 1.070)	0.167 *	(0.031, 0.887)
Family cohesion	0.694 ***	(0.569, 0.846)	0.545 *	(0.297, 1.000)
Social-related				
Rehabilitation assistance	1.495	(0.803, 2.784)	1.797	(0.291, 11.083)
Policy satisfaction	0.815	(0.604, 1.099)	1.382	(0.695, 2.747)
Social attitude	1.027	(0.752, 1.404)	0.726	(0.309, 1.701)
Barrier-free construction	0.742 *	(0.568, 0.970)	0.401 *	(0.185, 0.869)

ORs were adjusted for non-modifiable variables: caregiver’s age, gender, child’s age, gender, and disability type. Marital status (0: married, 1: others); Education (1: junior high school and below, 2: senior high school, 3: junior college and above); Employment status (0: not employed, 1: part-time/full-time employed); Caregiver’s comorbidities (0: no, 1: yes); Disability severity (1: mild, 2: moderate, 3: moderate-severe, 4: severe); Child’s comorbidities (0: no, 1: yes); Sleep problem (0: no, 1: yes); Mood swing (1–5: never-always); Home participation (1–5: never-always); Community participation (1–5: never-always); House ownership (0: no, 1: yes); Family cohesion (1–5: extremely disharmonious-extremely harmonious); Rehabilitation assistance (0: no, 1: yes); Policy satisfaction (1–5: extremely dissatisfied-extremely satisfied); Social attitude (1–5: extremely unfriendly extremely friendly); Barrier-free construction (1–5:extremely dissatisfied-extremely satisfied). * *p* < 0.05. ** *p* < 0.01. *** *p* < 0.001.

## Data Availability

Data cannot be shared publicly because of the privacy implications but is available upon reasonable request. Data requests may be sent to the corresponding author.
